# Pregnancy-associated breast cancers are driven by differences in adipose stromal cells present during lactation

**DOI:** 10.1186/bcr3594

**Published:** 2014-01-09

**Authors:** Jessica McCready, Lisa M Arendt, Eugene Glover, Vandana Iyer, Jerrica L Briendel, Stephen R Lyle, Stephen P Naber, Daniel G Jay, Charlotte Kuperwasser

**Affiliations:** 1 Department of Anatomy and Cellular Biology, Sackler School of Graduate Biomedical Sciences, Tufts University School of Medicine, 136 Harrison Ave, Boston, MA 02111, USA; 2 Molecular Oncology Research Institute, Tufts Medical Center, 800 Washington Street, Boston, MA 02111, USA; 3 Department of Cancer Biology, University of Massachusetts Medical School, 364 Plantation Street, Worcester, MA 01605, USA; 4 Department of Physiology, Sackler School of Graduate Biomedical Sciences, Tufts University School of Medicine, 136 Harrison Ave, Boston, MA 02111, USA; 5 Department of Pathology, Tufts Medical Center, 800 Washington Street, Boston, MA 02111, USA; 6 Current address: Department of Natural Sciences, Assumption College, 500 Salisbury Street, Worcester, MA 01609, USA

## Abstract

**Introduction:**

The prognosis of breast cancer is strongly influenced by the developmental stage of the breast when the tumor is diagnosed. Pregnancy-associated breast cancers (PABCs), cancers diagnosed during pregnancy, lactation, or in the first postpartum year, are typically found at an advanced stage, are more aggressive and have a poorer prognosis. Although the systemic and microenvironmental changes that occur during post-partum involution have been best recognized for their role in the pathogenesis of PABCs, epidemiological data indicate that PABCs diagnosed during lactation have an overall poorer prognosis than those diagnosed during involution. Thus, the physiologic and/or biological events during lactation may have a significant and unrecognized role in the pathobiology of PABCs.

**Methods:**

Syngeneic *in vivo* mouse models of PABC were used to examine the effects of system and stromal factors during pregnancy, lactation and involution on mammary tumorigenesis. Mammary adipose stromal cell (ASC) populations were isolated from mammary glands and examined by using a combination of *in vitro* and *in vivo* functional assays, gene expression analysis, and molecular and cellular assays. Specific findings were further investigated by immunohistochemistry in mammary glands of mice as well as in functional studies using ASCs from lactating mammary glands. Additional findings were further investigated using human clinical samples, human stromal cells and using *in vivo* xenograft assays.

**Results:**

ASCs present during lactation (ASC-Ls), but not during other mammary developmental stages, promote the growth of carcinoma cells and angiogenesis. ASCs-Ls are distinguished by their elevated expression of cellular retinoic acid binding protein-1 (crabp1), which regulates their ability to retain lipid. Human breast carcinoma-associated fibroblasts (CAFs) exhibit traits of ASC-Ls and express crabp1. Inhibition of crabp1in CAFs or in ASC-Ls abolished their tumor-promoting activity and also restored their ability to accumulate lipid.

**Conclusions:**

These findings imply that (1) PABC is a complex disease, which likely has different etiologies when diagnosed during different stages of pregnancy; (2) both systemic and local factors are important for the pathobiology of PABCs; and (3) the stromal changes during lactation play a distinct and important role in the etiology and pathogenesis of PABCs that differ from those during post-lactational involution.

## Introduction

The etiology and prognosis of breast cancer is complex with many factors contributing to both the lifetime risk of developing the disease and the aggressiveness of the disease once it is diagnosed. For example, breast cancers diagnosed in premenopausal women tend to be more aggressive than those diagnosed in postmenopausal women [1-3]. Likewise, pregnancy-associated breast cancers (PABCs), those diagnosed during pregnancy, lactation, or in the first postpartum year, are typically found at an advanced stage, have a higher incidence of lymph node metastases and are poorly differentiated [4-13].

Several hypotheses have been proposed to explain the etiology and pathobiology of PABCs. One hypothesis suggests that the elevated levels of circulating hormones present during pregnancy act on cancer cells to increase their biologic aggressiveness [4,13-15]. A second hypothesis suggests that the hormonal changes present during and following pregnancy increase vascularization and inflammatory cell recruitment, which together contribute to the adverse outcomes associated with PABCs [4,13,16].

Experimental support for the latter model comes from recent studies showing that implantation of cancer cells into the mammary glands of mice undergoing involution leads to accelerated tumor formation and metastasis through increased inflammation, matrix remodeling and angiogenesis [16-19]. Such experiments have emphasized the importance of involution on PABCs despite the fact that significant epidemiological data indicate that breast cancers diagnosed during lactation exhibit the most aggressive traits and an elevation in cause-specific death [10,13,15,20,21]. This association could not be explained by or adjusted for age, extent of disease or pregnancy hormones. Thus, the physiologic and/or biological events unique to lactation but not to other stages of pregnancy may have a significant and unrecognized role in the pathobiology of aggressive PABCs.

Lactation is a stage of mammary gland development associated with epithelial terminal differentiation and milk production. This stage is also associated with significant changes to the vasculature, the adipose tissue and the extracellular matrix [22,23]. During lactation, adipocyte and stromal remodeling is recognized for its importance in meeting the nutritional and metabolic demands of the expanding epithelium as well as in providing paracrine endocrine functions that are necessary for proper milk production [22,23]. Thus, it is highly plausible that these changes may be co-opted to meet the nutritional and metabolic needs of an expanding carcinoma leading to aggressive features; however, whether this is the case and whether it plays a role in PABCs is unclear.

In this study, we sought to study mammary stromal remodeling during development to examine its role in PABCs. Using a well-defined *in vivo* model of mammary gland development, we discovered that adipose tissue stromal cells (ASCs) present in the mammary gland during lactation provide novel insights into the phenotypic and functional diversity of stromal cells in development and reveal their importance in the pathogenesis of PABCs.

## Methods

### Cell lines and tissue culture

The 4T1-12B cell line [24] was obtained from Dr. Gary Sahagian (Tufts University), while the COMMA D cell line [25] was obtained from Dr. Daniel Medina (Baylor University) and the SUM159 cell line was obtained from Dr. Stephen Ethier (Kramanos Institute). NIH3T3 and 3T3-L1 cells were purchased from ATCC (Manassas, Virginia, USA). 4T1-12B, Comma D, NIH-3T3, and cells were cultured in (D)MEM with 10% fetal bovine serum and 1% Penicillan/Streptomycin/Fungicide (P/S?F)/S/F. SUM159 cells were cultured in Ham’s F12 with 5% calf serum, insulin (5 μg/mL) and hydrocortisone (5 μg/mL). CAF-L cells were generated as previously described [26,27]. 3T3-L1 and CAF-L cells were cultured in (D)MEM supplemented with 10% calf serum. All cells were grown at 37°C and 5% CO_2_. All cell lines were tested negative for mycoplasma (MilliPROBE, Millipore, Billerica, MA, USA); however, the identity of each cell line was not authenticated in our laboratory.

### Animals and surgery

All animal procedures were conducted in accordance with a protocol approved by the Tufts University Institutional Animal Care and Use Committee (IACUC). Colonies of Balb/c and NOD/SCID mice were maintained in-house. NOD/SCID mice were maintained under aseptic sterile conditions. All mice were given food and water *ad libitum*. Surgeries were performed under sterile conditions, and animals received antibiotics in the drinking water for two weeks after all surgical procedures.

**Figure 1:**
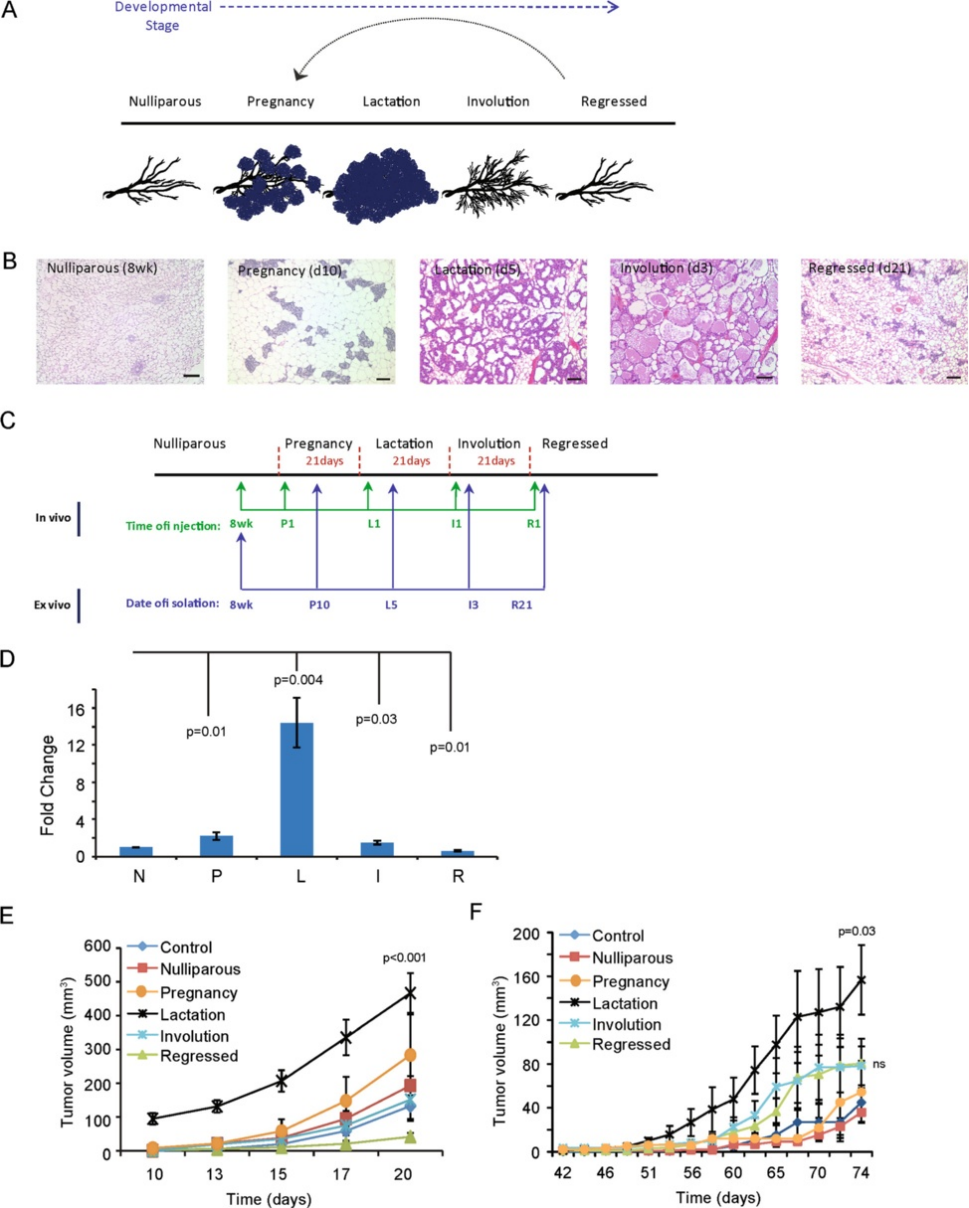
**Developmental stage of the mammary gland adipose tissue influences breast cancer growth. (A)** Schematic of stages of postnatal mammary gland development. **(B)** Representative H & E-stained sections of mammary glands isolated at five stages of development. Scale bar = 100 μm. **(C)** Diagram depicting experimental strategy and timepoints of *in vivo* mammary gland injections and adipose tissue isolation. See Methods for specific details. **(D)** 4 T1-12B cells were injected into the fourth inguinal fat pad of Balb/c mice at each developmental timepoint (n = 10 per group). Tumor weight was normalized to the nulliparous group. **(E)** The tumor growth curve of mice injected at eight weeks (nulliparous) of age with 4 T1-12B cells co-mixed with mouse adipose stromal cells (n = 10 per group). *P* values indicate significance compared to nulliparous mice. Data are means ± SEM. **(F)** Tumor growth curve of mice injected at eight weeks of age with COMMA-D cells co-mixed with mouse adipose stromal cells (n = 10 per group). *P* value indicates significance compared to nulliparous. Data are means ± SEM. SEM, standard error of the mean.

**Figure 2:**
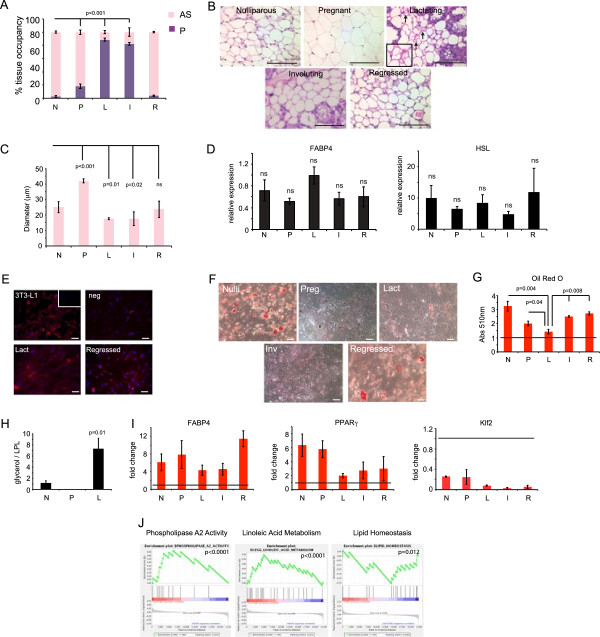
**Phenotypic modulation of adipocytes during mammary gland development. (A)** Quantification of percent area per tissue occupied by adipose stroma (AS) or parenchyma (P) using Spot software (n = 5 glands per stage). *P* values indicate significance compared to nulliparous. **(B)** Representative H & E-stained sections of mammary gland adipocytes isolated from non-diseased mice. Arrows indicate lipolytic adipocytes. **(C)** Average diameter (μm) of adipocytes. n = 3 glands per stage. *P* value indicates significance compared to nulliparous. **(D)** Quantitative RT-PCR of FABP4 and HSL expression in adipose stromal cells. Data are presented as average 2^(-ΔCt)^ ± SEM; n = 3 experiments. **(E)** Representative immunofluorescence images of adipose stromal cells isolated from lactating or regressed mammary gland and NIH3T3 cells (negative control), 3T3L1 cells (positive control) with inset (no primary) for FABP4 (red) and counterstained for nuclei with DAPI (blue) (n = 3 per group). **(F)** Representative images of Oil Red O staining and quantification **(G)** of adipose stromal cells. Line indicates untreated cells whose absorbance is set to one; n = 3 experiments. **(H)** Quantification of glycerol secreted by adipose stromal cells normalized to lipoprotein lipase RNA levels. n = 3 experiments. **(I)** Quantitative RT-PCR of Klf2, PPARγ, FABP4 in adipose stromal cells treated with adipocyte differentiation media. Data are presented as fold change in comparison to undifferentiated cells. Horizontal black line indicates undifferentiated cells set to one. Bars above the black line indicate an increase in expression compared to undifferentiated cells. Bars below the black line indicate a reduction in expression compared to undifferentiated cells. Data are presented as average 2^(-ΔΔCt)^ ± SEM. n = 3 experiments. **(J)** Gene set enrichment analysis of transcriptional signature of adipose stromal cells from lactating mice. Scale bar = 100 μm. All data are means ± SEM. DAPI, 4′,6-diamidino-2-phenylindole; SEM, standard error of the mean.

For developmental studies (Figure 1C; Figure 2A-C), the fourth inguinal mammary glands were removed from eight-week old female Balb/c mice (nulliparous). For all other developmental stages, eight-week old female Balb/c mice were mated with eight-week old male Balb/c mice and males were removed after the mice tested positive for vaginal plugs. Glands were isolated from pregnant mice 10 days after vaginal plugs were present. Following parturition, pups were allowed to nurse for five days at which time lactating glands were isolated or ten days at which time pups were removed to induce synchronous mammary gland involution. Involuting mammary glands were isolated three days after pups were removed and regressed mammary glands were isolated 21 days after pup removal.

For tumor studies (Figure 1D-F), mice were monitored weekly until palpable tumors formed, and tumor growth was measured and recorded using calipers at least once per week thereafter. For direct injections (Figure 1D), female Balb/c mice were mated at eight-weeks of age and injected into the fourth inguinal mammary gland. Due to the large number of mice needed per group and the coordination required to have all the mice developmentally synchronized, not all groups were injected at the same time. Rather, for each developmental stage, a nulliparous cohort control group was injected in parallel on the same day as a particular developmental stage with identical 4 T1-12B cells. This strategy was employed to control for the different injection dates for each cohort and any possible differences in the biology of the same 4 T1-12B cells on a given day. 4 T1-12B cells were resuspended in 3:1 media/Matrigel solution and injected at a total of 5,000 cells per gland on the first day of each developmental stage. Since we intentionally utilized a tumor model that forms tumors in approximately three weeks, and pregnancy, lactation, and involution are all naturally approximately 21 days (three weeks) in the mouse [28], one is able to compare the effects of the developmental stages to tumorigenesis. Tumors were extracted and weighed at 21 days. For co-mix experiments (Figure 1E-F) cells were resuspended in 3:1 media/Matrigel solution (5,000 4 T1-12B : 750,000 adipocytes; 250,000 Comma D : 750,000 adipocytes or 250,000 SUM159 : 750,000 CAF-L) and injected orthotopically into eight-week old nulliparous female Balb/c mice or eight-week nulliparous female NOD/SCID mice. Mice were monitored until palpable tumors formed, and tumor growth was measured and recorded using calipers two times a week thereafter. All tissues were embedded in either paraffin or optimal cutting temperature (OCT) compound, sectioned and stained for H & E at Tufts Medical Center.

### Primary tissue isolation and culture

All human breast tissue procurement for these experiments was obtained in compliance with the laws and institutional guidelines, as approved by the Institutional Review Board (IRB) committee from Tufts Medical Center and University of Massachusetts Medical School. Primary breast tumor tissues were obtained at Tufts Medical Center and University of Massachusetts Medical School. Disease-free breast tissues were obtained from discarded material of patients undergoing elective reduction mammoplasty at Tufts Medical Center and, therefore, informed consent is not required. Breast tissues were minced and enzymatically digested overnight with a mixture of collagenase and hyaluronidase as previously described [28,29]. Large clusters of undigested tissue were allowed to settle and the supernatant enriched for the adipocyte and stromal vascular factions. Cells were collected, washed and plated in serum containing medium to enrich for mammary adipose stromal cells (ASCs or carcinoma-associated fibroblasts (CAFs)). Cells were cultured in (D)MEM supplemented with 10% calf serum and 1% P/S/F for less than five passages.

Adipose stromal cells were isolated from mouse adipose tissue; it was divided into stromal and adipocyte fractions as described previously [30]. Briefly, freshly excised tissue from the fourth inguinal subcutaneous fat pads of Balb/c mice, was rinsed in PBS, minced and digested for 45 minutes to 1 hour at 37°C in collagenase and hyaluronidase. The fat pads were isolated from eight-week old female mice (nulliparous), or female mice that were mated and collected during pregnancy, lactation, involution, or following regression as described above. The floating adipocyte fraction and the stromal-vascular fraction were resuspended in (D)MEM and plated with 10% fetal bovine serum and 1% P/S/F. All *in vivo* and *in vitro* experiments with adipose tissue were performed with low passage cells (<3 passages).

### Differentiation assays

The adipocyte differentiation assay was conducted as follows: 100,000 low passage adipose stromal cells were plated into six-well dishes and allowed to grow to confluence in (D)MEM supplemented with 10% fetal bovine serum and P/S/F. Two days post confluence the cells were treated with differentiation media ((D)MEM supplemented with 10% fetal bovine serum, P/S/F, 0.5 mM IBMX (Sigma, St. Louis, MO, USA), 0.1 μM dexamethasone (Sigma) and 0.5 μg/mL insulin (Sigma) three times a week for 21 days. Cells were stained with Oil Red O, which was extracted and quantified as previously described [31]. Images were visualized using a Nikon Eclipse 80i inverted microscope and captured with Spot Imaging software (Diagnostic Instruments, Sterling Heights, MI, USA).

### Immunohistochemistry and immunofluorescence

LYVE-1 (1:100; Abcam, Cambridge, MA, USA) immunohistochemistry was performed on formalin-fixed, paraffin-embedded tissue sections with sodium citrate antigen retrieval, followed by detection with biotinylated secondary antibody (1:200 anti-rabbit; Vector Laboratories) and visualization with the Elite ABC Peroxidase Kit and DAB substrate (Vector Laboratories, Burlingame, CA, USA). Nuclei were counterstained for hematoxylin. Immunofluorescence on frozen tissue or cultured cells was performed on samples that were permeabilized with 0.1% Triton X-100 (Sigma) in PBS, washed and blocked in 1% BSA in PBS at ambient temperature. Samples were incubated with cellular retinoic acid binding protein-1 (crabp1) (1:250, Abcam), CD31 (1:100, Invitrogen, Carlsbad, CA, USA), pan-cytokeratin (1:100, DAKO, Carpinteria, CA, USA)) or FABP4 (1:200, Cell Signaling Technology, Danvers, MA, USA) overnight at 4°C. The fluorescence signal was detected using secondary antibodies (1:500 conjugated Alexa488 and Alexa588, Invitrogen). Nuclei were stained with 4′,6- diamidino-2-phynylindole (DAPI) and images were captured with the Spot imaging software system (Diagnostic Instruments, Inc.). Quantification was performed using ImageJ software. Immunofluorescence on frozen tissue sections was performed as previously described for crabp1 [32].

### Endothelial cell assays and culture

Primary mouse heart endothelial cells were obtained from the Center for Vascular Biology Research at Beth Israel Deaconess Medical Center. Briefly, hearts were isolated from 17-day-old Balb/c pups, minced and enzymatically digested with collagenase. Endothelial cells were extracted in a two-step immunomagnetic bead separation, first with CD31-coated beads (Pharmingen, San Diego, CA, USA) then with ICAM-coated beads (Pharmingen). Purified endothelial cells were cultured in (D)MEM, 20% fetal bovine serum, P/S/F, L-gln, NEAA, heparin, and endothelial cell growth supplement (Biomedical Technologies, Stoughton, MA, USA).

The human, umbilical cord endothelia cell (HUVEC) line was obtained from Dr. Guo-fu Hu (Tufts Medical Center) and cultured in endothelial cell media (Invitrogen) with 5% fetal bovine serum and basic fibroblast growth factor (bFGF) (20 ng/mL).

Conditioned media were collected from low passage adipose stromal cells isolated from three separate mice or three independent patients. Assays using mouse or human conditioned media were conducted identically.

In the proliferation assay, 50,000 endothelial cells were plated in a 24-well plate in growth media. Six hours after plating, cells were washed and treated with conditioned media from adipocytes. Cells were counted 36 hours after exposure to conditioned media.

In the wound healing assay, 300,000 endothelial cells were plated in a 12-well plate. Six hours after plating cells were washed and refed with (D)MEM supplemented with 2% fetal bovine serum. Twenty-four hours after plating, cells were washed with PBS, scratched with a pipet tip, treated with conditioned media and imaged (0 hour timepoint). Cells were imaged again six hours later. Wound closure was calculated using Spot software.

In the tube forming assay, 30,000 endothelial cells were plated onto a 96-well plate coated with Matrigel and treated with conditioned media from stromal cells. Five hours after plating, images were captured.

### Microarray

Total RNA was extracted from low passage adipose stromal cultures isolated from nulliparous, lactating and involuting mammary glands of female Balb/c mice (Figure 1B) with the RNAeasy Mini Kit (Qiagen, Valencia, CA, USA). Synthesis of cDNA from total RNA and hybridization/scanning of microarrays were performed with Affymetrix (Santa Clara, CA, USA) GeneChip products (Affy Mouse Gene 1.0 ST) as described in the GeneChip manual. Raw data files (.CEL) were converted into probe set values by Robust multi-array average normalization. Robust multi-array average expression values were computed from arrays using R and Bioconductor software. After normalization, genes not detected on all of the arrays and those with little variation (<0.05) in the signal across the arrays were removed from further consideration. To compare gene expression between different tissue phenotypes, linear models were derived using LIMMA, also part of the Bioconductor package. The significance levels of the comparisons were estimated by empirical Bayes methods. All microarray data can be accessed in the Gene Expression Omnibus (GEO) database using the accession number GSE53044.

For pathway analysis, gene set enrichment analysis was performed using the GenePattern gene set enrichment analysis (GSEA) module. Gene sets for the *mus musculus* genome were obtained from the Walter and Eliza Hall Institute (WEHI, Parkville VIC, Australia). Enriched gene sets with a false discovery rate (FDR) of less than 25% were identified as interesting. The normalized log 2 ratios of probes mapping to the same gene (defined by gene symbols) were averaged to generate independent expression estimates, and the resulting matrix data were median-centered.

### RT-PCR analysis

Total RNA was isolated and purified using a RNAeasy kit (Qiagen). RNA was reverse transcribed to cDNA using an iScript cDNA synthesis kit (Biorad, Hercules, CA, USA). Quantitative real-time PCR analysis was performed using SyBR Green and an iCycler thermocycler (Bio-Rad) and analyzed via the delta delta Ct method [33-35]. Primer sequences used for quantitative real-time PCR are listed in Additional file 1.

### Lipolysis assay

A total of 1,000 low passage ASCs were plated in a 96-well plate, allowed to grow to confluence for one week and then treated with differentiation media for three weeks. Glycerol levels were measured using the 3 T3-L1 lipolysis assay kit according to the manufacturer’s protocol (Zen-Bio, Research Triangle Park, NC, USA).

### Tumor necrosis quantification

Hematoxylin and eosin staining was performed on formalin-fixed, paraffin embedded tumor sections and necrosis was quantified across the entire tumor tissue using ImageJ software. Percent necrosis was calculated as the area of necrosis divided by the total area of the tumor.

### Statistical analysis

For the microarray, significance levels of the comparisons were estimated by empirical Bayes methods. All other analyses were done using a two-tailed Student’s t test.

## Results

### Local and systemic factors during lactation promote breast cancer

To study PABCs *in vivo*, we utilized a model we previously described [36] in which carcinoma cells are introduced directly into the fourth inguinal mammary glands of mice at the five major stages of postnatal development: post-pubertal (nulliparous), pregnancy, lactation, involution and upon full regression of the gland following involution (Figure 1A-C). To limit overlap of tumor growth across different developmental stages, we utilized a syngeneic model of triple negative basal like breast cancer that rapidly forms primary mammary carcinomas within three weeks (Figure 1C).

Consistent with earlier findings [16,19], 4 T1-12B carcinoma cells formed larger tumors in involuting mammary glands relative to nulliparous glands (*P* = 0.03) (Figure 1D). However, tumors harvested from pregnant and lactating mice were on average 3.5-fold larger than their counterparts in nulliparous mice (201 mg versus 58 mg; *P* <0.01) and 2-fold larger than in involuting mice (201 mg versus 98 mg; *P* = 0.045) (Figure 1D, Additional file 2). These results suggest that factors present during pregnancy and lactation contribute more strongly to tumor growth than during involution. Due to the aggressive nature of the 4 T1-12B model, no obvious histopathological differences were observed between 4 T1-12B tumors that formed between the different stages of mammary gland development.

Circulating levels of estrogen and progesterone are at their highest during pregnancy, while the levels of prolactin, a potent mitogen for breast cancer cells, are at their peak during pregnancy and lactation [37,38]. Since these hormones are powerful modulators of mammary epithelial cell proliferation, the accelerated breast tumor growth during pregnancy and lactation could be due to differences in systemic hormone levels. An alternative possibility is that local alterations in cellular composition and phenotypes within the mammary gland microenvironment could be sufficient to promote the observed increase in tumor growth during pregnancy and lactation. To evaluate the latter possibility, we co-mixed 4 T1-12B cells with the ASCs isolated from mice at each developmental stage and injected the co-mixture into nulliparous mice. Since nulliparous mice have comparable systemic hormone profiles, any differences in tumor growth would be attributable to the admixed stromal cells.

Although injection into mammary glands of pregnant, lactating and involuting mice accelerated tumor formation in the experiments above (Figure 1D), only ASCs derived from lactating mammary glands could accelerate 4 T1-12B mammary tumor growth in nulliparous mice (*P* <0.001, Figure 1E). To determine whether the difference in tumor growth rates might be associated with differential cell survival following implantation, injection sites of early 4 T1-12B tumors were evaluated and viable cells were observed within the mammary glands one week following injection, suggesting that the delay in early tumor growth was not due to immune clearance of cells or increased cell death. To test whether the tumor-enhancing effects of ASCs derived from lactating mammary glands extended to other breast epithelial models, we repeated the above experiment with COMMA-D cells, which are weakly to non-tumorigenic and when transformed also model triple negative basal like breast cancers [39]. While COMMA-D cells did not form large tumors when injected alone, they did grow slowly when co-mixed with ASCs from nulliparous, pregnant, involuting or regressed mammary glands. There was a trend towards faster growth in the tumors co-mixed with ASCs isolated from pregnant and involuting glands; however, this trend was not statistically significant. In contrast to ASCs from other stages of development, co-mixture with ASCs from lactating glands resulted in significant acceleration of tumor formation by COMMA-D cells (*P* = 0.03, Figure 1F). These findings indicate that while systemic factors likely play a key role in the development of tumors during pregnancy and involution, local alterations in either adipose stromal cell composition or function during lactation strongly contribute to breast cancer growth.

### Characterization of the adipose stroma during mammary gland development

The proportion of the mammary gland occupied by stroma is known to vary significantly depending on the differentiation state of the adjacent epithelium [22]. We, therefore, undertook a detailed histological and cellular analysis of the adipose stroma across the different stages of post-natal development to characterize stromal adipocyte remodeling during different mammary epithelial differentiation stages. Quantification of adipocyte occupancy revealed that they occupy the largest proportion of the gland in nulliparous and regressed glands (97.2% and 96.1%, respectively), while adipocytes comprise the smallest proportion of the gland during lactation (11.4%, *P* <0.001, Figure 2A). Adipocytes present in nulliparous and regressed tissues were variably sized and were both multilocular and unilocular (Figure 2B). In contrast, adipocytes present in pregnant mice were uniquely unilocular and significantly larger in size, consistent with a lipid storage phenotype (*P* <0.001, Figure 2C). Notably, adipocytes present in lactating tissues were markedly distinguishable from adipocytes in any other developmental stage. Not only were lactation-associated adipocytes significantly smaller in size (*P* = 0.01) but, more importantly, they exhibited features consistent with lipolysis [40] including prominent membrane folding and vesicle formation (Figure 2B arrows).

To examine further the mammary stroma, ASCs were isolated from mammary fat pads of several mice at different developmental stages. Although ASCs consist of heterogeneous populations of cell types including adipocyte stem cells, preadipocytes, mature adipocytes and other stromal cells, we found that the ASCs from all developmental stages isolated from three different mice all expressed similar levels of mature adipocyte markers including FABP4 and HSL (Figure 2D,E) indicating that the representation of mature adipocytes within the cultures was similar. However, when examined for their ability to accumulate and store lipid, Oil Red O staining revealed that ASCs from lactating glands failed to accumulate lipid while ASCs from nulliparous, pregnant, involuting and regressed glands all induced significant lipid accumulation under identical conditions (Figure 2F,G).

Since the lactating-derived ASCs failed to accumulate lipid, we reasoned this might be due to (1) the ability to retain lipid (lipid metabolism) differed between ASCs isolated from different developmental stages, (2) the induction of adipogenic differentiation differed between ASCs isolated from different developmental stages, or (3) there were significant differences in the representation of cell types within ASCs isolated from different developmental stages that could affect lipid accumulation. To evaluate these possibilities, we assayed ASCs from mammary glands of nulliparous, pregnant, lactating and regressed mice for lipolyis, adipogenesis and cellular composition.

Lipid metabolism is maintained by the highly regulated balance of lipogenesis and lipolysis [41]. During lipid catabolism, lipids are broken down into glycerol and free fatty acids, a process that can be measured biochemically. We quantified the levels of glycerol released from ASCs under differentiation conditions and compared them to the expression level of lipoprotein lipase, an enzyme required for lipogenesis [42]. ASCs isolated from lactating mammary glands exhibited a significantly higher ratio of lipolysis to lipogenesis compared to nulliparous- and pregnant-derived cells (*P* = 0.01, Figure 2H, Additional file 3).

Adipocyte differentiation involves a stepwise program triggered by the suppression of Klf2 expression with the concomitant induction of PPARγ and FABP4 [43]. We observed that ASCs isolated from all developmental stages, including lactation, were able to activate PPARγ and FABP4 expression and repress Klf2 expression in response to differentiation conditions (Figure 2I). However, compared to ASCs from other stages, the magnitude of PPARγ and FABP4 induction in ASCs from lactating and involuting mammary glands was reduced, suggestive of an attenuated, although not blocked, differentiation potential of adipocytes.

We examined ASC cultures for the presence of other contaminating mesenchymal cell types that could affect adipogenesis including preadipocytes (Pref1), endothelial cells (CD31), angioblasts (CD34), mesenchymal stem cells (Sca-1 and CD29) and macrophages (F4/80). However, no significant differences were observed between ASCs derived from lactating mammary glands compared to any of the other developmental stages that could account for their failure to undergo adipogenesis or lipid accumulation (Additional file 4). Furthermore, gene-expression profiling of ASCs from nulliparous, involuting and lactating mammary glands from multiple mice indicated that very few genes were differentially expressed (Additional files 5, 6 and 7). GSEA further revealed that ASCs from lactating mammary glands were highly enriched in genes associated with adipocytes including linoleic acid metabolism (*P* <0.0001), phospholipase A2 activity (an enzyme that releases fatty acids from glycerol, *P* <0.0001) and lipid homeostasis (Figure 2J, *P* = 0.012). Together, these results suggest that ASCs from lactating mammary glands (hereafter termed lactation-derived adipose cells (ASC-Ls)) are enriched in adipocytes that are lipolytic, unable to accumulate lipid and are unable to fully execute the adipogenic differentiation program.

### Tumor-promoting functions of ASC-Ls

**Figure 3:**
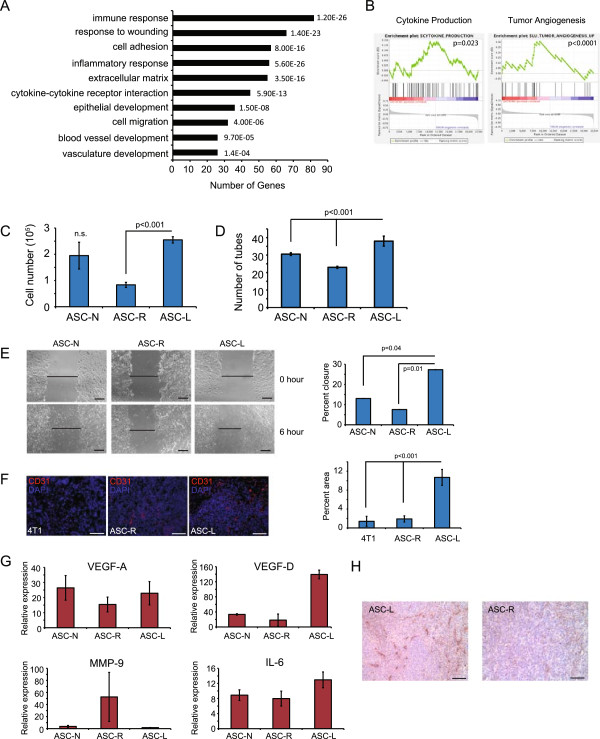
**ASC-Ls promote tumors through increased vasculogenesis. (A)** Gene ontology biological process categories associated with adipose stromal cells from lactating mice compared to mammary adipose stromal cells from nulliparous or involuting mice. The DAVID Functional Annotation Tool was used to define categories with an enrichment score >1.5, and the number of genes represented in the list and the *P* value of genes differentially expressed in the microarray are shown. **(B)** Gene set enrichment analysis indicates that ASC-Ls exhibit increased expression of genes associated with cytokine production and tumor angiogenesis. **(C-E)** Endothelial cell assays using primary mouse heart endothelial cells treated with conditioned media collected from ASCs isolated from nulliparous, lactating or regressed glands (ASC-N, ASC-L or ASC-R, respectively), n = 3 per group. Data are presented as means ± SEM **(C)** Proliferation assay day 3. Data are presented as means ± SEM. n = 3 experiments. **(D)** Quantification of tube formation assay four hours after plating. Data are presented as means ± SEM. n = 3 experiments. **(E)** Representative image and quantification of wound healing assay six hours after addition of conditioned media. Data are presented as means ± SEM. n = 3 experiments. **(F)** Representative immunofluorescence and quantification of 4 T1-12B tumors co-mixed with adipocytes for CD31 (red) and counterstained for nuclei with DAPI (blue) (n = 4 per group). Data presented as means ± SEM. **(G)** Quantitative RT-PCR of VEGF-A, VEGF-D, MMP-9 and IL-6 in ASC-N, ASC-R or ASC-L. Data are presented as average 2^(-ΔCt)^ ± SEM; n = 3 experiments. **(H)** Representative images of immunohistochemistry of 4 T1-12B tumors co-mixed with adipocytes for LYVE-1. n = 3 experiments. Scale bar = 100 μm. ASC-L, lactation-derived adipose stromal cells; ASC-N, nulliparous-derived adipose stromal cells; ASC-R, regressed-derived adipose stromal cells; DAPI, 4′,6-diamidino-2-phenylindole; MMP-9, matrix metalloproteinase 9; SEM, standard error of the mean; VEGF, vascular endothelial growth factor.

To determine the functional properties of ASC-Ls their gene expression signature was queried for known biological process by GSEA. ASC-Ls were enriched in genes involved in extracellular matrix production, immune/inflammatory response and cytokine-cytokine receptor interactions compared to nulliparous-derived adipose cells (ASC-Ns) or involuting derived adipose (ASC-Is) cells (Figure 3A, 3B; Additional files 8, 9, 10 and 11). In addition, compared to ASC-Ns or regressed-derived adipose cells (ASC-Rs), ASC-Ls were highly enriched in the gene set termed ‘tumor angiogenesis’ (*P* <0.0001) and, to a lesser degree, ‘angiogenesis’ (Figure 3A, 3B; Additional files 8, 9 and 10). This finding prompted us to assess whether the tumor promoting activity of ASC-Ls might be through angiogenesis.

Primary mouse heart endothelial cells (MHECs) were treated with conditioned medium (CM) harvested from ASC-Ns, ASC-Rs or ASC-Ls and examined for proliferation, wound healing and tube formation. Increased proliferation of MHECs was observed following stimulation with CM from ASC-Ls (*P* <0.001) compared to cells treated with CM from ASC-Rs (Figure 3C). MHEC tube formation was also significantly augmented in the presence of CM from ASC-Ls compared to CM from ASC-Ns or ASC-Rs (*P* <0.001, Figure 3D, Additional file 12A). Furthermore, wound healing assays revealed that MHECs migrated significantly faster when treated with CM from ASC-Ls when compared to CM from ASC-Ns or ASC-Rs (*P* = 0.01, Figure 3E).

To determine if the increased angiogenic and inflammatory activity of ASC-Ls was contributing to their ability to accelerate tumor formation, 4 T1-12B cells were co-mixed with ASC-Rs or ASC-Ls and injected into nulliparous mice. Macrophage recruitment and tumor angiogenesis was assessed by immunofluorescence (IF). No significant difference was found in the number of F4/80 positive macrophages recruited to tumors derived from 4 T1-12B cells alone, 4 T1-12B cells co-mixed with ASC-Ls or 4 T1-12B cells co-mixed with ASC-Rs (Additional file 12B). However, there was a 10-fold increase in the number of CD31 positive endothelial cells present within tumors derived from 4 T1-12B cells co-mixed with ASC-Ls (*P* <0.001, Figure 3F) compared to tumors from 4 T1-12B cells alone or from 4 T1-12B cells co-mixed with ASC-Rs.

Given these findings, the expression of secreted pro-angiogenic growth factors implicated in angiogenesis (vascular endothelial growth factor-A, -C, -D (VEGF-A, VEGF-C, VEGF-D), matrix metalloproteinase-9 (MMP-9), IL-6, placental-like growth factor (PlGF) and platelet-derived growth factor (PDGF-C)) was examined in ASC-Ls (Figure 3G and Additional file 12C). Only the levels of VEGF-D and IL-6 were significantly elevated in ASC-Ls compared to ASC-Ns or ASC-Rs. Consistent with the function of these factors, tumors derived from 4 T1-12B cells co-mixed with ASC-Ls showed an increase in Lyve-1 staining (a marker of lymphatic endothelial cells) compared to tumors derived from 4 T1-12B cells co-mixed with ASC-Rs (Figure 3H). Collectively, these results indicate that ASC-Ls accelerate tumor growth through their increased angiogenesis.

### Phenotypic modulation of ASC-Ls is regulated by crabp1

**Figure 4:**
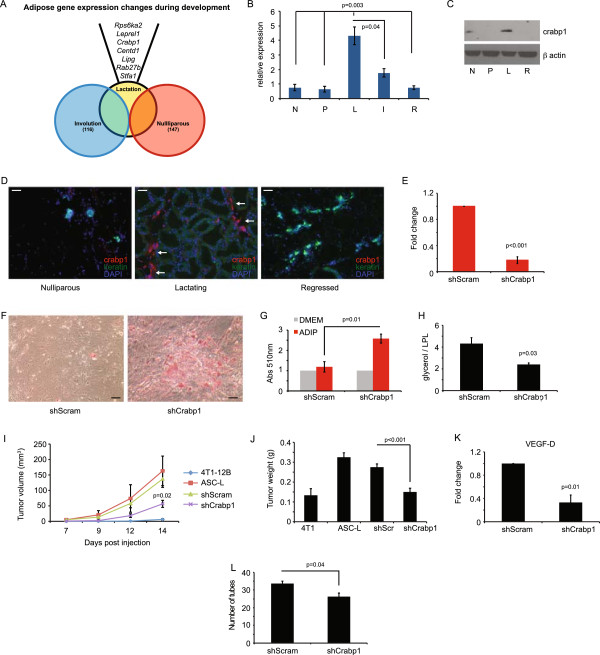
**Phenotypic modulation of adipocytes is regulated by Crabp1. (A)** Overlap of differentially regulated genes in adipocytes. **(B)** qRT-PCR of crabp1 transcript in adipocytes isolated from the fourth inguinal mammary gland. (N = nulliparous, P = pregnant, L = lactation, I = involution, R = regressed). Data are presented as average 2^(-ΔCt)^ ± SEM; n = 3 experiments. **(C)** Western blot of crabp1 protein in adipocytes as in **(B)**. **(D)** Immunofluorescence of crabp1 (red) and cytokeratin (green) in mammary glands; DAPI nuclear counterstain (blue). Arrows indicate adipocytes that are crabp1 positive. Scale bar = 100 μm. **(E)** qRT-PCR of crabp1 transcript in ASC-L treated with shRNA for crabp1 or scrambled control. Data are presented as average 2^(-ΔΔCt)^ ± SEM; n = 3 experiments. **(F)** Representative images of Oil Red O staining of ASC-L transduced with shRNA (crabp1 or scrambled control) and exposed to adipocyte differentiation media. **(G)** Quantification of Oil Red O staining from **(F)**. Data are presented as average absorbance ± SEM. Untreated cell absorbance is set to one; n = 3 experiments. **(H)** Quantification of glycerol secreted by ASCs normalized to lipoprotein lipase RNA levels. n = 3 experiments. **(I)** Tumor growth curve and tumor weight **(J)** of mice injected at eight weeks with 4 T1-12 B cells co-mixed with untreated ASC-L or ASC-L transduced with shRNA (crapb1 or scrambled control) (n = 10 per group). *P* value indicates significance compared to shScram-ASC-L. Data are means ± SEM. **(K)** qRT-PCR of VEGF-D in ASC-Ls transduced with shRNA for crabp1 or scrambled control. Data are presented as average 2^(-ΔΔCt)^ ± SEM; n = 3 experiments. **(L)** Quantification of tube formation of primary mouse heart endothelial cells treated with media collected from ASC-Ls transduced with shRNA (crabp1 or scrambled control). Data are presented as means ± SEM. n = 3 experiments. ASC-L, lactation-derived adipose stromal cells; crabp1, cellular retinoic acid binding protein-1; DAPI, 4′,6-diamidino-2-phenylindole; SEM, standard error of the mean.

To determine how ASC-Ls are regulated, we conducted analyses of the ranked genes that were differentially expressed between ASC-Ls, ASC-Ns and ASC-Rs. In doing so, we found that crabp1 was one of the most significantly differentially expressed genes in ASC-Ls compared to ASC-Ns or ASC-Rs (Figure 4A). Crabp1 has been well studied for its role as a binding protein for retinoic acid [44,45], but its role in regulating lipid metabolism or adipocyte differentiation is less well understood [46].

In agreement with the microarray analysis, we confirmed that crabp1 mRNA and protein levels were highly expressed in ASC-Ls (Figure 4B,C). Crabp1 mRNA was overexpressed approximately five-fold in ASC-Ls (L) compared to any other developmental stage (N, P, R: *P* = 0.003; I: *P* = 0.04). Likewise, crabp1 protein was most abundantly expressed in ASC-Ls. Crabp1 expression was also examined within mammary tissues from different developmental stages by IF (Figure 4D). Abundant levels of crabp1 protein expression were found localized within adipocytes in mammary glands during lactation (arrows) but not in adipocytes from mammary glands of nulliparous or regressed mice.

To determine whether crabp1 expression is directly regulating the adipogenic phenotype of ASC-Ls, lentiviral-mediated short hairpin-inhibition was used to target crabp1 expression. Crabp1 gene expression was reduced by 86% when compared to cells treated with scrambled shRNAs (*P* = 0.01; Figure 4E). Crabp1 inhibition led to a dramatic increase in lipid accumulation under differentiation conditions (Figure 4F,G). Furthermore, inhibition of crabp1 expression resulted in a marked decrease in the levels of glycerol release relative to lipoprotein lipase expression from cultures of adipocytes upon differentiation compared to control ASC-Ls (Figure 4H, *P* = 0.03).

To determine whether phenotypic modulation though crabp1 was responsible for the tumor promoting ability of ASC-Ls, 4 T1-12B tumor cells were co-mixed with ASC-Ls in which crabp1 was inhibited and injected into nulliparous mice. Tumor cells co-mixed with shCrabp1-ASC-Ls grew significantly slower (*P* = 0.02, Figure 4I) and were significantly smaller (*P* <*0*.001, Figure 4J) than tumors derived from 4 T1-12B cells co-mixed with either control ASC-Ls or shScram-ASC-Ls. In addition a three-fold decrease in VEGF-D expression was observed upon inhibition of crabp1 expression when compared to shScram-ASC-Ls cells (*P* = 0.01, Figure 4K). Endothelial tube formation was also inhibited in endothelial cells treated with media isolated from shCrabp1-ASC-L cells (Figure 4L). Taken together, these findings indicate that the tumor promoting phenotype of ASC-Ls, which can be modulated by crabp1, is important for angiogenesis.

### Breast carcinoma-associated fibroblasts phenocopy ASC-Ls

**Figure 5:**
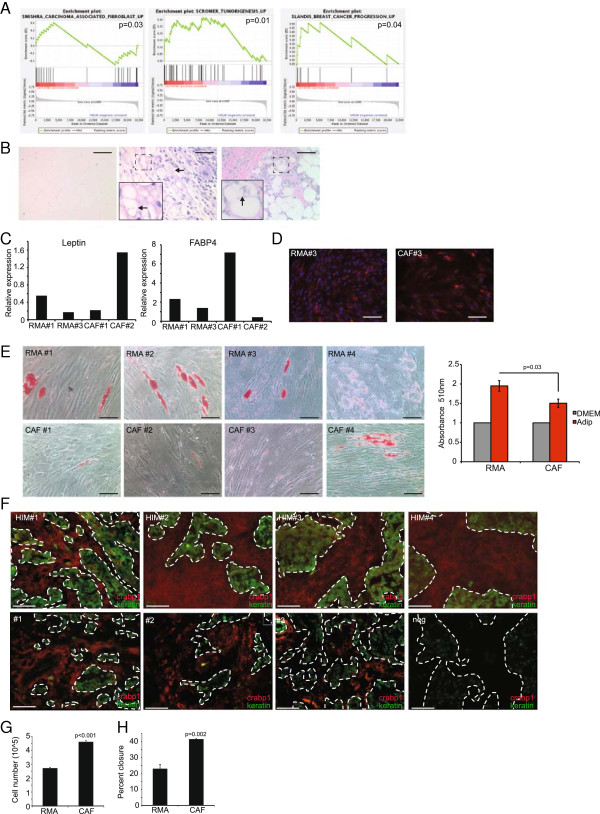
**CAFs phenocopy ASC-Ls. (A)** Gene sets enriched in ASC-Ls. **(B)** Representative H & E-stained sections of normal human mammary gland adipocytes (left), human mammary carcinoma (middle) and human xenograft breast cancer (right). Arrows and inset indicate prominent membrane folding. Scale bar = 100 μm. **(C)** Quantitative RT-PCR of leptin and FABP4 in RMA and CAFs from various patient samples. Data are presented as average 2^(-ΔΔCt)^ ± SEM. **(D)** Representative image of immunofluorescence of RMAs and CAFs for FABP4 (red) and counterstained for nuclei with DAPI (blue) (n = 3 per group). **(E)** Representative images of Oil Red O staining and quantification of adipose tissue from reduction mammoplasty (RMA) (n = 8) and carcinoma associated fibroblasts (CAF) (n = 6) treated with adipocyte differentiation media. Data are presented as average absorbance ± SEM. Untreated cell absorbance is set to one. **(F)** Immunofluorescence of human mammary carcinoma either as xenografts in the HIM model (n = 4) or primary human breast cancer tissues, n = 10. Crapb1 (red) cytokeratin (green) showing both positive (#1 to 3) and negative expression (neg). **(G-H)** Endothelial cell assays using HUVECs treated with conditioned media collected from RMAs or CAFs, n = 3 per group. Data are presented as means ± SEM. **(G)** Proliferation assay day 3. **(H)** Quantification of wound healing assay six hours after addition of conditioned media. Scale bar = 100 μm. ASC-L, lactation-derived adipose stromal cells; crabp1, cellular retinoic acid binding protein-1; DAPI, 4′,6-diamidino-2-phenylindole; HUVEC, human umbilical cord endothelial cells; SEM, standard error of the mean.

In examining the functional properties of ASC-Ls, GSEA revealed that ASC-Ls were unexpectedly enriched in genes expressed in CAFs (Figure 5A, *P* = 0.03). Additionally, an enrichment of genes associated with tumorigenesis (*P* = 0.01) and breast cancer progression (*P* = 0.04) was also found in ASC-Ls (Figure 5A and Additional files 13, 14 and 15). These findings suggest that CAFs may share similarities with ASC-Ls and likewise be regulated by the same mechanisms that regulate ASC-Ls. Indeed, consistent with their known angiogenic functions [47], CAF-CM significantly stimulated both the proliferation (*P* < 0.001, Figure 5G) and migration (*P* = 0.002, Figure 5H) of HUVECs compared to control cells isolated from disease-free reduction mammoplasty tissues.

When examined for features of ASC-Ls including elevated crabp1expression, expression of adipocyte markers, features of lipolysis and a failure to undergo adipogenic differentiation, CAFs exhibited remarkable similarities to ASC-Ls. Compared to the normal adipose stroma of disease-free human breast tissue (Figure 5B left), the adipocytes adjacent to human xenografted tumors (Figure 5B middle) or adjacent to breast carcinomas in patient tumors (Figure 5B right) frequently exhibited features of lipolytic adipocytes including membrane blebbing and decreased adipocyte diameter. Isolated CAFs from breast tumor specimens (n = 6) were examined for the expression of adipocyte markers and lipid accumulation and compared to adipose cells isolated in the identical manner from reduction mammoplasty adipose (RMA, n = 8). Notably, CAFs expressed leptin and FABP4, two markers of mature adipocytes (Figure 5C, D), yet failed to accumulate lipid and undergo adipogenesis compared to RMAs (*P* = 0.03, Figure 5E).

Immunofluoresence of orthotopic xenograft breast tumors revealed that crabp1 expression was absent in tumor cells (green) but highly expressed within the desmoplastic fibroblastic tumor-associated stroma (red) in all samples tested (Figure 5F, Additional file 16). Crabp1 was also expressed within the tumor-associated stroma of patient-derived primary human breast cancer tissues (Figure 5F, Additional files 16, 17 and 18).

### Phenotypic modulation of CAFs is regulated by crabp1

**Figure 6:**
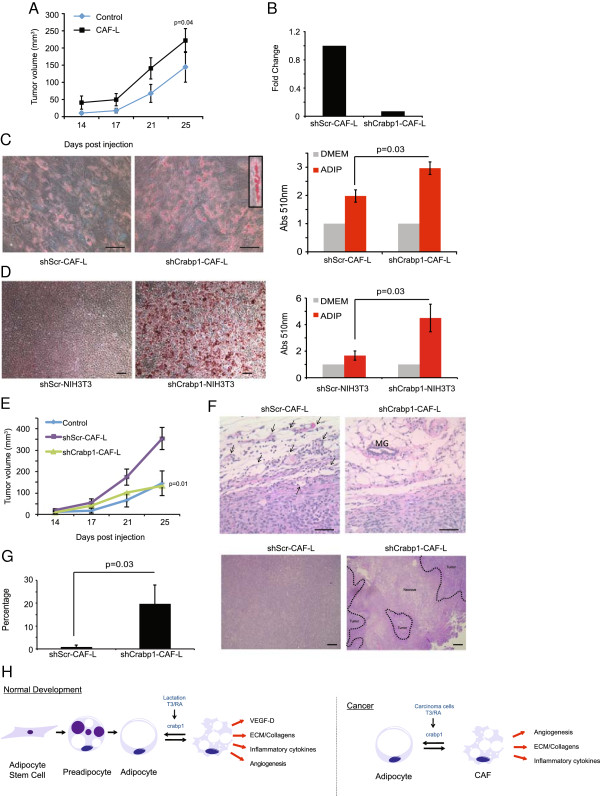
**Crabp1 regulates CAF phenotype. (A)** Tumor growth curve of mice injected at eight weeks of age with SUM159 cells co-mixed with CAF-L cells (n = 10 per group). *P* value indicates significance compared to SUM159 cells alone. Data are means ± SEM. **(B)** Quantitative RT-PCR of crabp1 in CAF-L cells transduced with shRNA for crabp1 or scrambled control. Data are presented as average 2^(-ΔΔCt^ ± SEM; n = 3. **(C)** Representative image of Oil Red O staining and quantification of CAF-L cells transduced with shRNA for crabp1 or scrambled control and exposed to adipocyte differentiation media. Data are presented as average absorbance ± SEM. Untreated cell absorbance is set to one; n = 3 experiments. Scale bar = 100 μm. **(D)** Representative image of Oil Red O staining and quantification of NIH3T3 cells treated with shRNA for crabp1 or scrambled control and exposed to adipocyte differentiation media. Data are presented as average absorbance ± SEM. Untreated cell absorbance is set to one; n = 3 experiments. Scale bar = 100 μm. **(E)** Tumor growth curve of mice injected at eight weeks of age with SUM159 cells co-mixed with CAF-L cells transduced with shRNA for crapb1 or scrambled control (n = 10 per group). *P* value indicates significance compared to shScr-CAF-L cells. Data are means ± SEM. **(F)** Representative H & E images of SUM159 tumors co-mixed with CAF-L cells. V = vessel; MG = mammary gland duct. Scale bar = 100 μm. **(G)** Representative images and quantification of the percentage of centralized tumor necrosis in SUM159 cells co-mixed with CAF-L cells transduced with shRNA for crapb1 or scrambled control (n = 5 per group). **(H)** Schematic of phenotypic modulation of adipocytes under physiologic (normal development) and pathologic (breast cancer progression) conditions. CAF-L, carcinoma-associated fibroblast-like; crabp1, cellular retinoic acid binding protein-1; SEM, standard error of the mean.

Since these above findings indicate that CAFs exhibit many of the molecular and functional features of ASC-Ls, we wanted to determine whether the expression of crabp1 could also regulate the tumor promoting phenotype of CAFs. To this end, we generated CAF-like (CAF-L) cells as previously described [26], since we were unable to genetically manipulate primary CAFs due to their limited lifespan in culture. Consistent with prior findings [26], CAF-L cells exhibited many of the features of primary CAFs including the ability to accelerate tumor growth (*P* = 0.04, Figure 6A).

Inhibition of human crabp1 in CAF-L cells using lentiviral-mediated short hairpin led to a reduction of crabp1 gene expression by 96% compared to cells infected with control shRNA (shScr) (Figure 6B). Notably, inhibition of crabp1 in CAF-L cells led to increased intracellular lipid accumulation (*P* = 0.03, Figure 6C). To determine if phenotypic modulation of fibroblasts was restricted to CAF-L cells, we also inhibited crabp1 expression in NIH3T3 fibroblasts, which express abundant levels of crabp1 (Additional file 19). Similar to the findings in CAF-L cells, knockdown of crabp1 in NIH3T3 cells led to a dramatic induction of adipogenesis and accumulation of lipid under differentiation conditions (*P* = 0.03, Figure 6D).

To determine whether the phenotypic switch of CAFs affects their ability to promote tumor growth, the Basal B SUM159 breast cancer cell line was co-mixed with either shScr-CAF-L or shCrabp1-CAF-L cells and injected into NOD-SCID mice. Indeed, phenotypic modulation of CAF-L cells abolished their tumor promoting activity (Figure 6E, *P* = 0.01). This failure to accelerate SUM159 xenografts was attendant with a significant reduction in the density of the tumor-associated vasculature (Figure 6F) and resulted in extensive and significant regions of centralized tumor necrosis, which was essentially absent in tumors from SUM159 cells co-mixed with shScr-CAF-L cells (Figure 6G, *P* = 0.03). Since tumor necrosis is a well-established feature of limited angiogenesis, these findings, combined with those above, support the notion that crabp1 expression affects the tumor promoting activities of CAFs by modulating, in part, tumor angiogenesis.

## Discussion

It is well known that pregnancy and lactation confer long term protective effects in lowering the lifetime risk of developing breast cancer [48-50]. However, approximately 3% of women are diagnosed with breast cancer during pregnancy and lactation [13], and PABCs are more aggressive in nature compared to age-adjusted breast cancers within this age group [10,13,15,20,21]. Thus, a better understanding of the etiology and pathogenesis of PABCs is imperative for improved diagnosis and therapeutic strategies for pregnant and lactating women.

The systemic and microenvironmental changes during post-lactational involution have been best recognized for their role in the pathogenesis of PABCs [16,17,19,51,52]. In particular, studies have elucidated that the main drivers of tumor promotion in post-lactational involution are due to changes to the extracellular matrix as well as an infiltration of inflammatory macrophages. Our data demonstrate that the microenvironment of lactation may also play a role in progression of PABC. Significant epidemiological data indicate that PABCs diagnosed during lactation have an overall poorer prognosis than those diagnosed during involution [10,13,15,20,21]. However, these studies do not dissect the role of lactation specifically, by comparing breastfeeding and non-breastfeeding women. Further epidemiologic studies in these populations would clarify the relationship between lactation and involution in tumor promotion.

Consistent with the clinical observations, we found that breast cancer growth was significantly augmented when carcinoma cells were introduced into mammary glands of pregnant or lactating mice compared to cells injected into involuting mice. Our study is the first to assess the role of all stages of pregnancy on PABCs (Figure 1D-F). Data presented herein indicate that the lactating microenvironment is a strong driver of tumor progression suggesting that if a pre-existing tumor were present in the breast at the time of lactation, the lactating microenvironment could promote that tumor to be more aggressive than if the tumor were present during other stages of pregnancy. Our data show that ASCs present during lactation were the major affecters of breast tumorigenesis during this stage. It is important to note that lactating ASCs have never been implicated as a potential cause/promoter of increased aggressiveness. It also should be noted that these cells were isolated from non-diseased tissues yet they still have the ability to promote tumors when compared to any other stage of mammary gland development. These findings imply that both systemic and local factors are important for the pathobiology of PABCs and that the stromal changes during lactation under normal physiological conditions play a distinct and important role in the progression of PABCs that are different from those during post-lactational involution.

We found that lactating ASCs express increased levels of inflammatory cytokines, are highly angiogenic and are growth promoting. Furthermore, we found that expression of crabp1 in lactating ASCs was responsible for their failure to retain lipid and undergo adipogenesis. It is interesting that the abundance of such cells and their expression of crabp1 appear to be restricted to lactation and cancer. This suggests that the appearance of these ASCs may be important during conditions of high nutritional demand where they perform important functions to meet those demands (Figure 6H). Indeed, during pregnancy and lactation, the mammary gland undergoes a notable expansion in parenchyma when additional energy is required for cellular proliferation and expansion of the tissue (Figure 2). Likewise, during breast cancer growth, expanding carcinoma cells require further energy and angiogenic needs. By regulating lipogenesis and lipolysis, adipocytes can maintain the appropriate energy balance necessary to meet the nutritional demands of an expanding normal or malignant epithelial cell population [53]. Our findings suggest that CAFs may have reactivated the same pro-angiogenic, inflammatory, growth-promoting program that is present during lactation. Under developmental and pathological conditions, this program exists to satisfy the demands placed on the growing tissue. However, during breast cancer, activation of this adipose stromal developmental program is co-opted to further fuel tumor growth.

Our data indicate that by regulating adipogenesis and lipid metabolism, ASCs can acquire angiogenic and tumor growth promoting characteristics. Reduction in the levels of crabp1 in CAF-like cells not only shifted lipid metabolism towards lipid accumulation, but it also abolished tumor promoting activity. Our findings and recent observations by others indicate that CAFs exhibit many features of ASCs including adipogenesis, lipid accumulation and expression of mature adipocyte markers, albeit at attenuated levels compared to ASCs from disease-free breast tissues [54]. Furthermore, we, along with others, have found that adipocytes adjacent to breast cancers exhibit features of lipolysis [55]. Indeed, adipocytes co-cultured with breast cancer cells have been shown to release their lipid [55] suggesting signals derived from cancer cells promote lipolysis. Such adipocytes, termed cancer-associated adipocytes (CAAs) also over-express matrix proteins and inflammatory cytokines [55]. These similarities, combined with our findings, suggest that adipocytes may be an important and major source of CAFs in breast tumors tissues through activation of crabp1 expression (Figure 6G).

We have found that crabp1 levels are induced and over-expressed in ASCs of lactating mammary glands and ASCs of cancers (CAFs). However, the upstream regulator of its expression in normal and pathological conditions is unclear. During embryonic development, the *crabp1* promoter is demethylated to induce gene expression in specific tissues [56], while in adult tissues, the promoter becomes inaccessible through chromatin modification to restrict its expression [57]. *Crabp1* can be activated by thyroid hormone (T3/T4) binding of the holo-thyroid hormone receptors/retinoid receptors that in turn bind to the thyroid response element (TRE) located approximately 1 kb upstream of the *crabp1* basal promoter. The recruitment of this complex to the *crabp1* promoter results in disassembly of the nucleosome covering the transcription initiation site, chromatin remodeling and histone acetylation which leads to a stable state of active gene expression [46]. T3 is required for lactation and its levels are the highest during this stage of development [58,59]. Likewise, T3 levels have been reported to be elevated in the serum of breast cancer patients [60,61]. Thus, T3 may be a key regulator of *crabp1* expression in the mammary adipose stroma (Figure 6G). Indeed, the metastable phenotype of ASCs could be observed even after removal of the cells from tissues indicating that the mechanism regulating crabp1 expression is likely epigenetic. Further studies will be needed to fully elucidate the mechanism regulating crabp1 expression and whether thyroid hormone directly modulates its expression *in vivo*.

## Conclusions

These findings imply that PABC is a complex disease and, in agreement with clinical data, suggest that the lactating microenvironment promotes aggressive tumors. Both systemic and local factors are important for the pathobiology of PABCs, and stromal changes during lactation play a distinct and critical role in the progression of PABCs that differ from those during post-lactational involution. In addition, these findings reveal a previously unrecognized and critical role of specialized adipose stromal cells during lactation that contributes to PABC pathobiology.

## Abbreviations

ASC: adipose stromal cells; ASC-I: adipose stromal cells derived from involuting mammary glands; ASC-L: adipose stromal cells derived from lactating mammary glands; ASC-N: adipose stromal cells derived from nulliparous mammary glands; ASC-P: adipose stromal cells derived from pregnant mammary glands; ASC-R: adipose stromal cells derived from regressed mammary glands; BSA: bovine serum albumin; CAF: carcinoma-associated fibroblast; CAF-L: carcinoma-associated fibroblast-like; CM: conditioned media; CRABP1: cellular retinoic acid binding protein 1; DAPI: 4′,6-diamidino-2-phenylindole; (D)MEM: (Dulbecco’s) modified Eagle’s medium; FABP4: fatty acid binding protein 4; GSEA: gene set enrichment analysis; H & E: hemotoxylin and eosin; HSL: hormone sensitive lipase; HUVEC: human umbilical cord endothelial cell; IL-6: interleukin 6; KLF2: Krupple-like factor 2; MHEC: mouse heart endothelial cell; MMP-9: matrix metalloproteinase 9; NOD/SCID: non-obese diabetic severe combined immunodeficient; PABCs: pregnancy-associated breast cancers; PBS: phosphate-buffered saline; PDGF–C: platelet-derived growth factor C; PlGF: placental-like growth factor; PPARγ: peroxisome proliferator-activated receptor gamma; Pref1: preadipocyte factor 1; P/S/F: Penicillan/Streptomycin, Fungicide; RMA: Reduction mammoplasty adipose; RT-PCR: reverse transcriptase polymerase chain reaction; Sca-1: stem cell antigen 1; SEM: standard error of the mean; shCrabp1: small hairpin CRABP1; shScr: small hairpin scrambled; TRE: thyroid response element; VEGF-A: vascular endothelial growth factor A; VEGF-C: vascular endothelial growth factor C; VEGF-D: vascular endothelial growth factor D.

## Competing interests

The authors declare that they have no competing interests.

## Authors’ contributions

JM conceived of the hypothesis, designed and performed the experiments and wrote the manuscript. LA performed some of the experiments and helped write the manuscript. EG performed the microarray and GSEA analyses. VI performed a portion of the human adipocyte differentiation assays. JB quantified cells in the involuting mammary glands and determined the percentage of necrotic areas. SL and SN provided human clinical tissue for analysis. DJ read and revised the manuscript. CK conceived of the hypothesis, directed the project and wrote the manuscript. All authors read and approved the final manuscript.

## Supplementary Material

Additional file 1: Table S13 Description of data: RT-PCR primer sequences. Click here for file

Additional file 2 **Developmental stage of host affects tumor formation.** Description of data: Tumor weight (g) of tumors directly injected into mice at specific developmental stages (n = 8 per group). Data are presented as means ± SEM. Click here for file

Additional file 3 **Mammary gland adipocytes undergo lipolysis.** Description of data: Quantification of glycerol secreted by adipose stromal cells derived from nulliparous (N), pregnant (P) and lactating (L) mammary glands. n = 3 experiments. Click here for file

Additional file 4 **Characterization of stromal lineage gene expression in ASCs.** Description of data: Quantitative PCR of endothelial (CD31, CD34), macrophage (F4/80), fibroblast (FSP1), mesenchymal stem cell (Sca-1, CD29), preadipocyte (Pref-1), and epithelial (CK18, CK14) cell markers. Data are presented as average 2^(-ΔCt)^ ± SEM; n = 3 experiments. Click here for file

Additional file 5: Table S1 Description of data: Affymetrix microarray analysis of the statistically significant changes between adipose stromal cells isolated from nulliparous and involuting mice. Click here for file

Additional file 6: Table S2 Description of data: Affymetrix microarray analysis of the statistically significant changes between adipose stromal cells isolated from nulliparous and lactating mice. Click here for file

Additional file 7: Table S3 Description of data: Affymetrix microarray analysis of the statistically significant changes between adipose stromal cells isolated from lactating and involuting mice. Click here for file

Additional file 8: Table S4 Description of data: Genes included in Linoleic Acid gene set used for GSEA analysis. Click here for file

Additional file 9: Table S5 Description of data: Genes included in Lipid Homeostasis gene set used for GSEA analysis. Click here for file

Additional file 10: Table S6 Description of data: Genes included in Cytokine production gene set used for GSEA analysis. Click here for file

Additional file 11: Table S7 Description of data: Genes included in Tumor Angiogenesis gene set used for GSEA analysis. Click here for file

Additional file 12 **Angiogenic phenotypes of ASC-Ls.** Description of data: **(A)** Representative images and quantification of tube formation assay four hours after plating in CM from ASCs from regressed (ASC-R) or lactating (ASC-L) mammary glands. Data are presented as means ± SEM. n = 3 experiments. **(B)** Representative immunofluorescence and quantification of 4 T1-12B tumors co-mixed with ASC-Rs or ASC-Ls for F4/80 (red) and counterstained for nuclei with DAPI (blue) (n = 4 per group). Data are presented as means ± SEM. **(C)** Quantitative RT-PCR of VEGF-C, PDGFc, and PlGF in adipose stromal cells isolated from nulliparous, regressed and lactating mammary glands. Data are presented as average 2^(-ΔCt)^ ± SEM; n = 3 experiments. Click here for file

Additional file 13: Table S8 Description of data: Genes included in Carcinoma Associated Fibroblast gene set used for GSEA analysis. Click here for file

Additional file 14: Table S9 Description of data: Genes included in Tumorigenesis gene set used for GSEA analysis. Click here for file

Additional file 15: Table S10 Description of data: Genes included in Breast Cancer Progression gene set used for GSEA analysis. Click here for file

Additional file 16 **Human breast cancers express crabp1.** Description of data: Enlarged images of immunofluorescence of human mammary carcinoma either as xenografts in the HIM model (n = 4) or primary human breast cancer tissues, n = 10. crapb1 (red) cytokeratin (green) showing both positive (#2-3) or negative expression (neg). Click here for file

Additional file 17: Table S11 Description of data: Clinical characteristics of human breast cancer samples. Click here for file

Additional file 18: Table S12 Description of data: Clinical characteristics of human fibroblast samples. Click here for file

Additional file 19 **NIH3T3 cells express high levels of crabp1 protein.** Description of data: Western blot of crabp1in adipocytes isolated from the fourth inguinal mammary gland. Click here for file
