# Supplementary figures and images for: Pregnancy-associated breast cancers are driven by differences in adipose stromal cells present during lactation
Source: Breast Cancer Res. 2014 Jan 9;16(1):R2. doi: 10.1186/bcr3594 (PMC3978436; doi:10.1186/bcr3594)

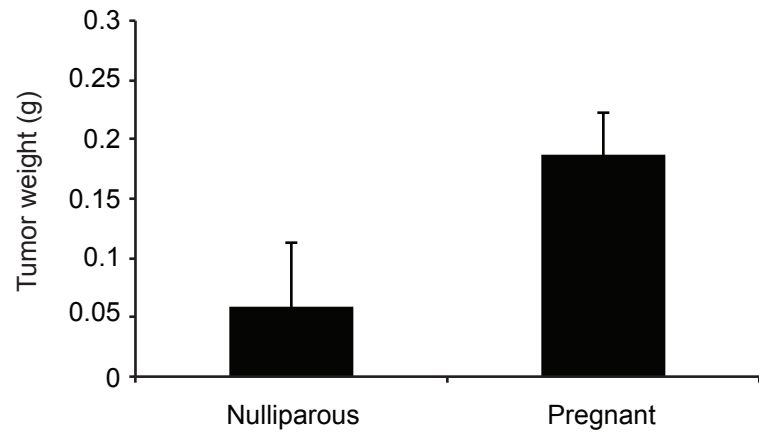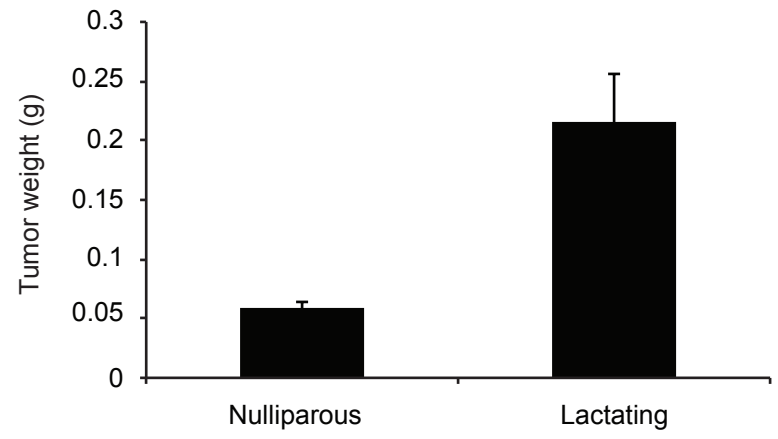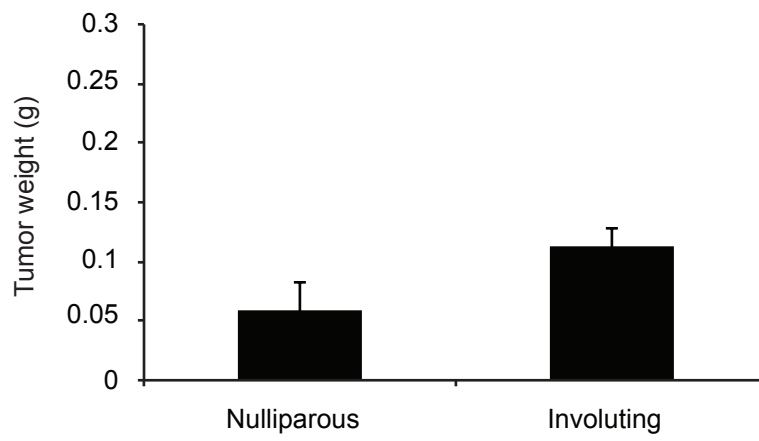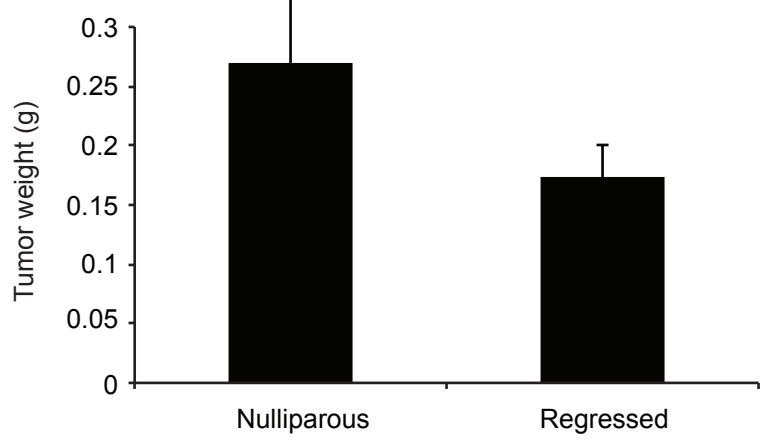

Supplement: Additional file 2 — Developmental stage of host affects tumor formation. Description of data: Tumor weight (g) of tumors directly injected into mice at specific developmental stages (n = 8 per group). Data are presented as means ± SEM. [file bcr3594-S2.pdf]

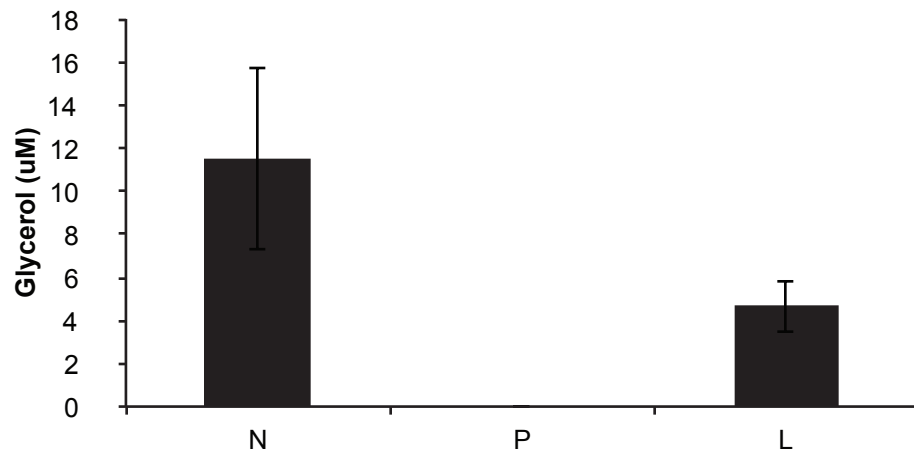

Supplement: Additional file 3 — Mammary gland adipocytes undergo lipolysis. Description of data: Quantification of glycerol secreted by adipose stromal cells derived from nulliparous (N), pregnant (P) and lactating (L) mammary glands. n = 3 experiments. [file bcr3594-S3.pdf]

A

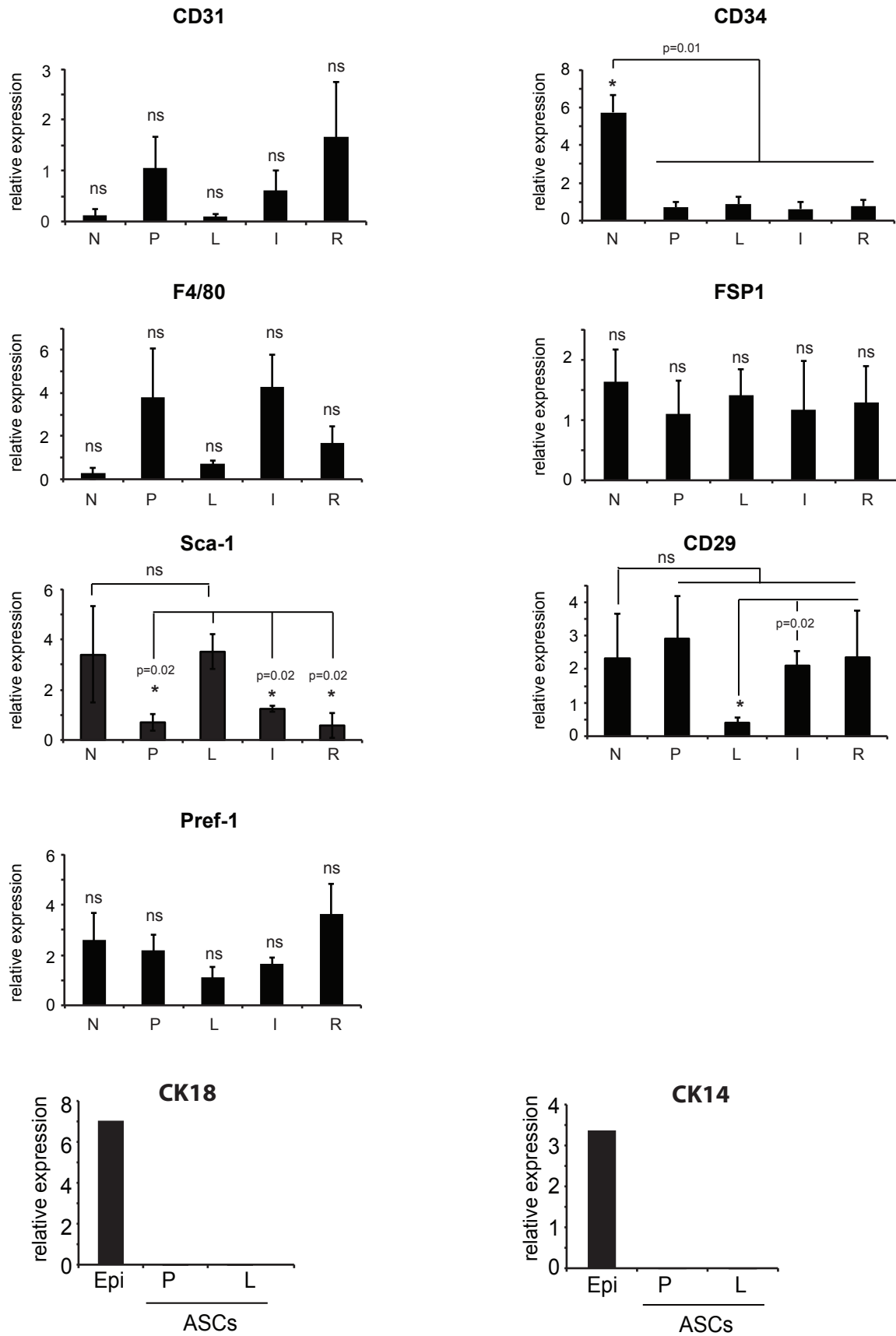

Supplement: Additional file 4 — Characterization of stromal lineage gene expression in ASCs. Description of data: Quantitative PCR of endothelial (CD31, CD34), macrophage (F4/80), fibroblast (FSP1), mesenchymal stem cell (Sca-1, CD29), preadipocyte (Pref-1), and epithelial (CK18, CK14) cell markers. Data are presented as average 2(-ΔCt) ± SEM; n = 3 experiments. [file bcr3594-S4.pdf]

A

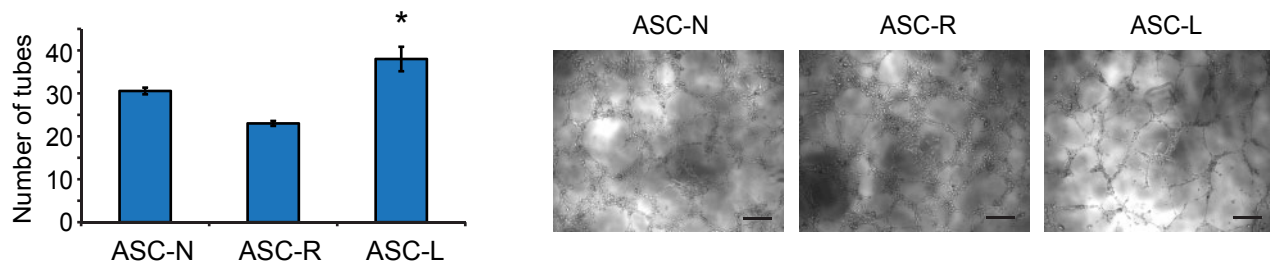

B

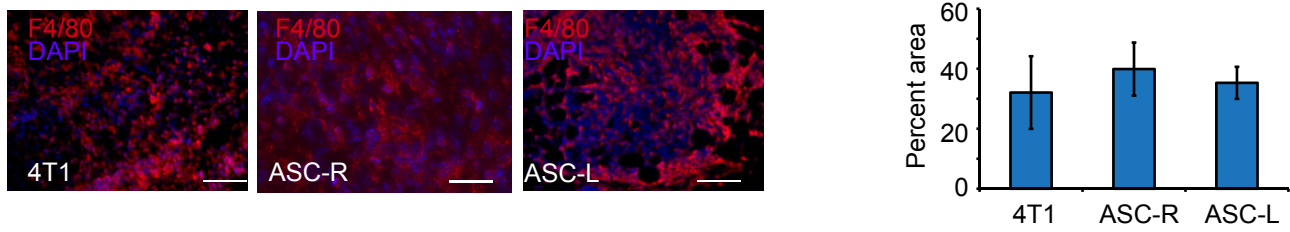

C

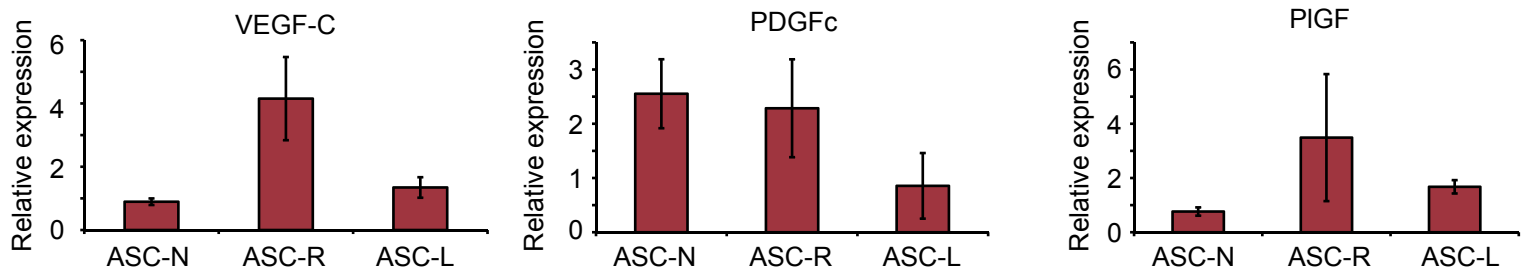

Supplement: Additional file 12 — Angiogenic phenotypes of ASC-Ls. Description of data: (A) Representative images and quantification of tube formation assay four hours after plating in CM from ASCs from regressed (ASC-R) or lactating (ASC-L) mammary glands. Data are presented as means ± SEM. n = 3 experiments. (B) Representative immunofluorescence and quantification of 4 T1-12B tumors co-mixed with ASC-Rs or ASC-Ls for F4/80 (red) and counterstained for nuclei with DAPI (blue) (n = 4 per group). Data are presented as means ± SEM. (C) Quantitative RT-PCR of VEGF-C, PDGFc, and PlGF in adipose stromal cells isolated from nulliparous, regressed and lactating mammary glands. Data are presented as average 2(-ΔCt) ± SEM; n = 3 experiments. [file bcr3594-S12.pdf]

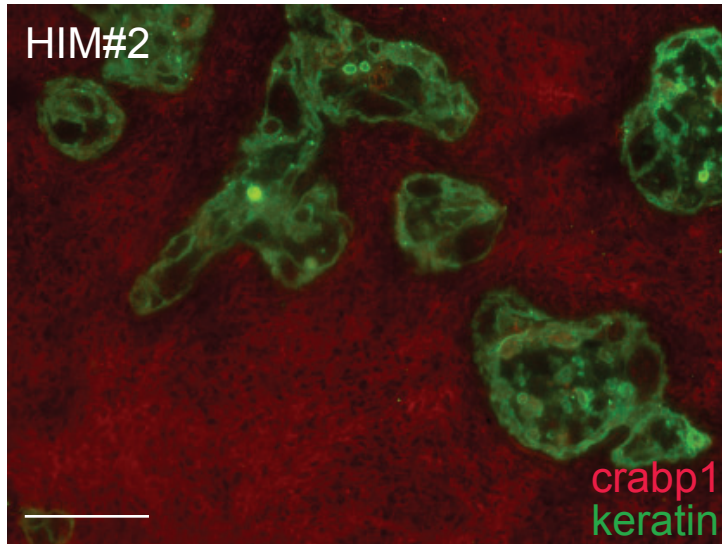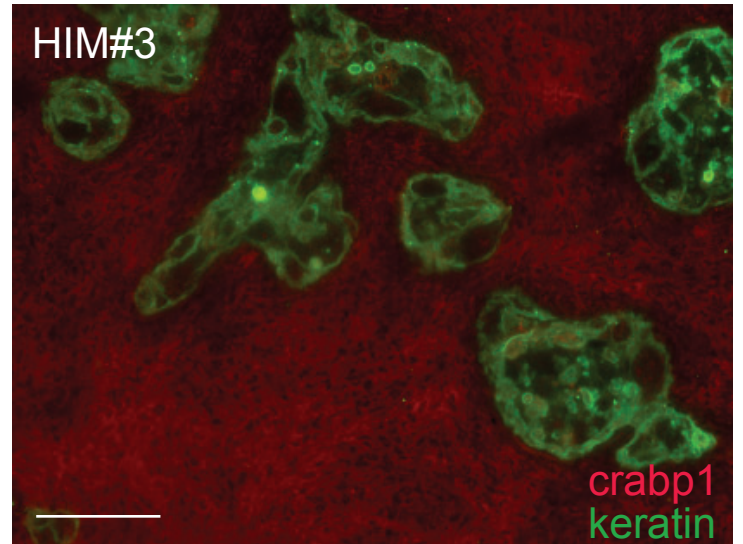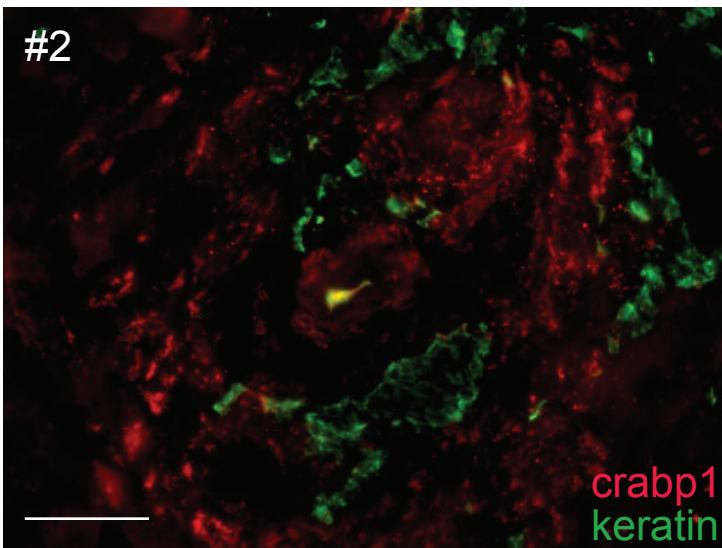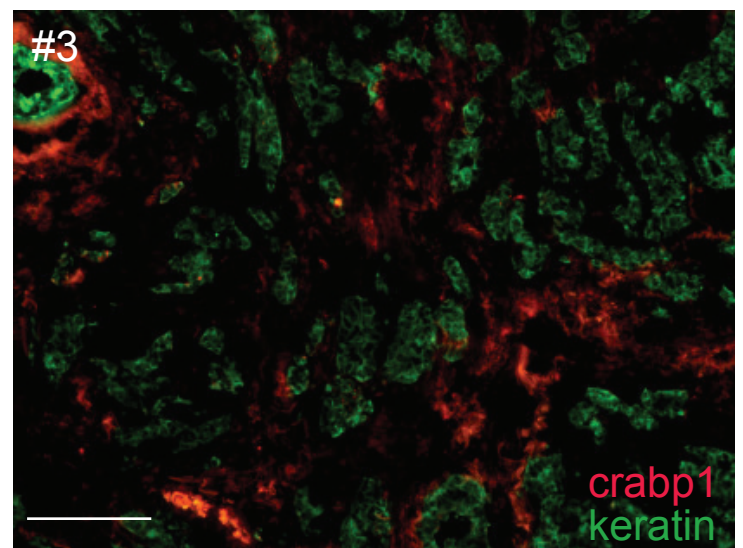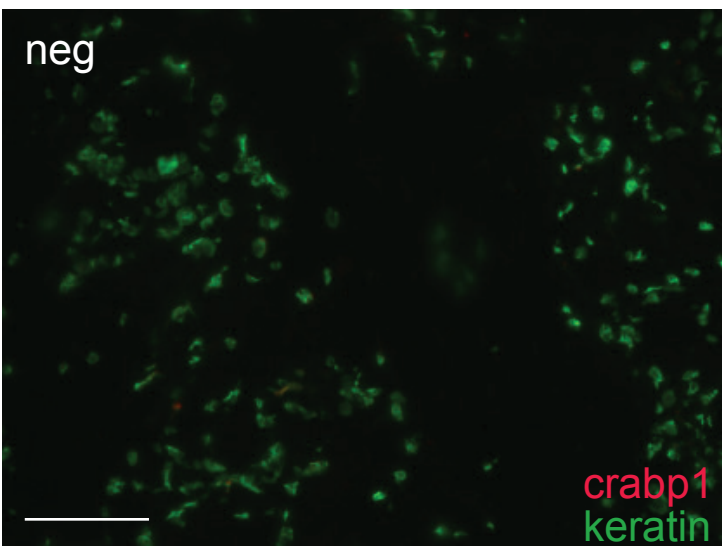

Supplement: Additional file 16 — Human breast cancers express crabp1. Description of data: Enlarged images of immunofluorescence of human mammary carcinoma either as xenografts in the HIM model (n = 4) or primary human breast cancer tissues, n = 10. crapb1 (red) cytokeratin (green) showing both positive (#2-3) or negative expression (neg). [file bcr3594-S16.pdf]

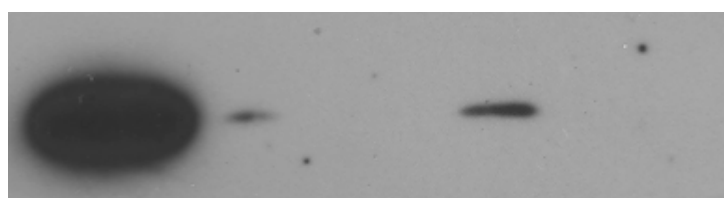

Crabp1

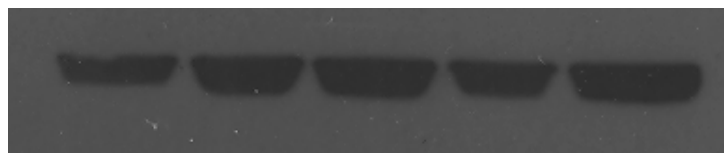

$\beta$  actin

NIH  
3T3

N

P

L

R

Supplement: Additional file 19 — NIH3T3 cells express high levels of crabp1 protein. Description of data: Western blot of crabp1in adipocytes isolated from the fourth inguinal mammary gland. [file bcr3594-S19.pdf]
